# Norfloxacin Derivative with Carbazole at C-7 FQB-1 Induces Cytotoxic, Antiproliferative, and Antitumor Effects in an Experimental Lung Carcinoma Model

**DOI:** 10.3390/ph18050664

**Published:** 2025-04-30

**Authors:** Alondra Bocanegra-Zapata, Hiram Hernández-López, Socorro Leyva-Ramos, Rodolfo Daniel Cervantes-Villagrana, Marisol Galván-Valencia, L. Angel Veyna-Hurtado, Norma Guadalupe Ramírez Tovar, Damaris Albores-García, Juan Armando Flores de la Torre, Alberto Rafael Cervantes-Villagrana

**Affiliations:** 1 Maestría en Ciencia y Tecnología Química, Unidad Académica de Ciencias Químicas, Universidad Autónoma de Zacatecas, Zacatecas 98160, Mexico; 36170547@uaz.edu.mx; 2 Laboratorio de Investigación en Síntesis y Modificación Química, Unidad Académica de Ciencias Químicas, Universidad Autónoma de Zacatecas, Zacatecas 98160, Mexico; hiram.hernandez.lopez@uaz.edu.mx; 3 Laboratorio de Investigación en Terapéutica Experimental, Unidad Académica de Ciencias Químicas, Universidad Autónoma de Zacatecas, Zacatecas 98160, Mexico; angelveyna@gmail.com (L.A.V.-H.); normarmzt33@gmail.com (N.G.R.T.); 4 Laboratorio de Investigación en Síntesis Orgánica, Facultad de Ciencias Químicas, Universidad Autónoma de San Luis Potosí, San Luis Postosí 78210, Mexico; sleyva@uaslp.mx; 5 Moores Cancer Center, University of California San Diego, La Jolla, CA 92093, USA; rdancervantes@gmail.com; 6 Department of Pharmacology, School of Medicine, University of California San Diego, La Jolla, CA 92093, USA; 7 Laboratorio de Neuropatología y Productos Naturales, Unidad Académica de Ciencias Químicas, Universidad Autónoma de Zacatecas, Zacatecas 98050, Mexico; gavm001144@uaz.edu.mx; 8 Departamento de Farmacobiología, Centro de Investigación y de Estudios Avanzados del Instituto Politécnico Nacional (Cinvestav), Unidad Sede Sur, Mexico City 14330, Mexico; dalbores@cinvestav.mx; 9 Laboratorio de Investigación en Toxicología y Farmacia, Unidad Académica de Ciencias Químicas, Universidad Autónoma de Zacatecas, Zacatecas 98160, Mexico; armando.flores@uaz.edu.mx

**Keywords:** fluoroquinolone, lung cancer, cytotoxic, antineoplastic, molecular docking

## Abstract

**Background:** Cancer remains a leading cause of morbidity and mortality worldwide. According to the World Health Organization (WHO), lung cancer is the most prevalent type of cancer among both men and women. Despite the various pharmacological and biological treatments available for lung cancer, their effectiveness has often fallen short, and the side effects can be severe. Therefore, there is an ongoing need to identify and develop novel compounds with enhanced anti-tumor efficacy and improved safety profiles. Research has shown that fluoroquinolone derivatives exhibit a broad cytotoxic spectrum comparable to other drugs used in clinical chemotherapy. **Objective:** The aim of this work was to synthesize and evaluate the cytotoxic, anti-proliferative, and anti-tumor effects of FQB-1, a novel fluoroquinolone derivative. **Results:** In silico molecular docking analysis demonstrated a strong interaction between FQB-1 and human topoisomerase, with a binding affinity score of –9.8 kcal/mol. In vitro cytotoxicity and anti-proliferative assays were conducted using the Lewis Lung Carcinoma (LLC) cell line. FQB-1 was tested at concentrations of 2.5, 5.0, 25.0, 50.0, 100.0, and 150.0 µg/mL. Significant cytotoxic and anti-proliferative effects were observed at concentrations of 50–150 µg/mL after 24 h of treatment. To evaluate FQB-1′s efficacy in vivo, a syngeneic tumor model was established in C57BL/6 mice. Treatment with FQB-1 (100 mg/kg) resulted in a marked reduction in tumor volume compared to the untreated control group. Histopathological analysis of tumor tissues from treated animals revealed a decrease in mitotic index and an increase in necrotic regions, indicating compromised tumor viability. **Conclusions:** FQB-1 exhibits cytotoxic and anti-proliferative effects and can reduce tumor growth while compromising tumor viability.

## 1. Introduction

Cancer has emerged as the leading cause of death worldwide and poses a significant public health challenge affecting both men and women [1]. Among the various types of cancer, lung cancer is the most lethal globally and ranks second in prevalence [2]. Despite the availability of treatment options [3], the overall incidence and mortality rates associated with lung cancer have not shown a significant decline [4]. Current standard treatment includes platinum-based chemotherapy in combination with topoisomerase inhibitors, such as etoposide. More recently, immune checkpoint inhibitors targeting Programmed Cell Death Protein 1 (PD-1) have been incorporated into treatment regimens [5].

Current cancer treatments remain limited by several factors, including high systemic toxicity, low selectivity, and the emergence of drug resistance mechanisms [6,7] collectively reducing the effectiveness of available therapeutic options. In this context, fluoroquinolones (FQ) have emerged as promising compounds with antitumoral potential, offering several therapeutic advantages such as reduced toxicity, less drug resistance, decreased risk of secondary tumors, and greater potency compared to traditional topoisomerases II inhibitors. These characteristics position fluoroquinolones as promising antitumor agents [8,9]. Mechanistically, the antitumor activity of FQs is primarily attributed to their ability to inhibit topoisomerase II, a critical enzyme involved in DNA replication and repair. This inhibition leads to the accumulation of double-stranded DNA breaks, ultimately triggering apoptotic pathways [10,11,12]. Given these mechanisms, fluoroquinolone derivatives represent a valuable chemical scaffold for the development of novel anticancer agents, either as stand-alone therapies or in combination with existing treatment regimens.

In the rational design and synthesis of novel FQ molecules, a structural modification at the C7 position could enhance the compound’s interaction with topoisomerase II, thereby improving the compound’s potency, spectrum of activity, and pharmacokinetic properties [13]. A second modification involves the incorporation of a carbazole moiety, a heterocyclic compound that has shown anticancer activity across several cancer cell lines [14]. Thus, it is expected that the substitution of the C7 position with a carbazole group increases the antitumor potential of the novel FQ compound. Additionally, a modification at the C3 position with a boryl difluoride group offers further pharmacological advantages. According to the WHO, boron is considered a “probable essential element” [15], and the FDA has approved several boron-containing drugs, reflecting their therapeutic value. These boron-based compounds have shown improved antimicrobial and anticancer properties, positively impacting medicinal chemistry [16,17].

The search for new molecules with therapeutic properties against cancer led us to design and synthesize a new norfloxacin derivative compound: “Difluoroboryl 1-ethyl-7-(1,2,3,4-tetrahydro-9*H*-carbazol-9-yl)-6-fluoro-4-oxo-1,4-dihydroquinoline-3-carboylate”, labeled as FQB-1. This work aimed to synthesize and evaluate the novel fluoroquinolone FQB-1 for its binding probability to topoisomerase in silico by molecular docking and to explore its cytotoxic effect on lung cancer cells and its anti-tumor therapeutic potential in an experimental syngeneic lung carcinoma model.

## 2. Results

### 2.1. Synthesis and Molecular Docking of FQB-1 Compound with IIα Human Topoisomerase

**Figure 1:**
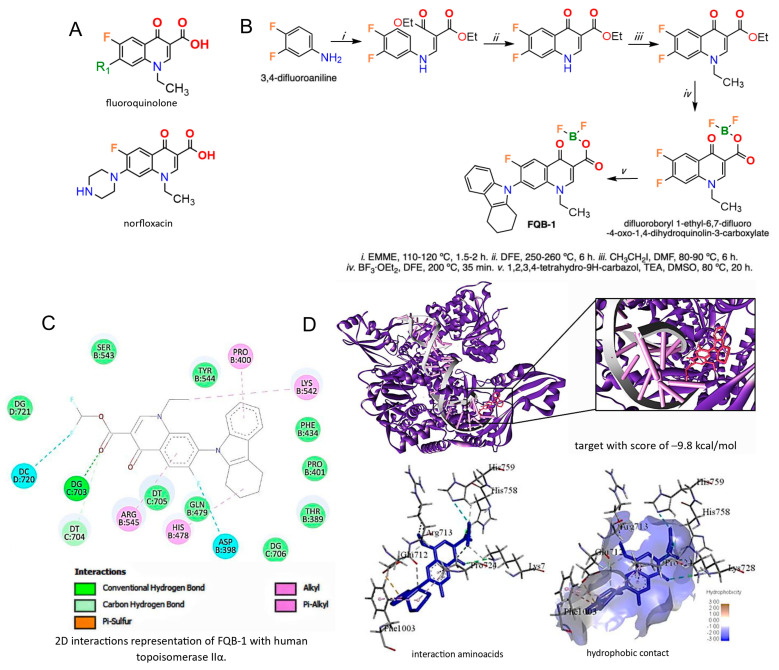
Synthesis and molecular docking of FQB-1 compound with IIα human topoisomerase. (**A**) Structure of fluoroquinolone and norfloxacin. (**B**) Synthesis of the FQB-1 compound from 3,4-difluoroaniline through a condensation reaction followed by an intramolecular thermocyclization to obtain 6,7-difluoro-4-oxo-1,4-dihydroquinoline-3-carboxylate ethyl, was finally subjected to a constant reflux and stirring system to generate the final compound. (**C**) Two-dimensional representation of the amino acids of IIα human topoisomerase (5GWK) with the chemical structure of the FQB-1 compound. (**D**) Interaction site between IIα human topoisomerase (5GWK) with the chemical structure of the FQB-1 compound represented in three dimensions, as well as the interaction amino acids and hydrophobic interactions.

FQ, represented by the two-ring core (Figure 1A, upper panel), serves as the basis for designing derivatives that improve the desired pharmacological properties, such as norfloxacin (Figure 1A, bottom panel). Based on the structural design of the FQB-1 compound, we began the molecular docking evaluation, which presented a score of −9.8 kcal/mol (Figure 1B–D). In this analysis, different types of interactions participated, including conventional-type interactions of hydrogen between guanine (DG:703) of the DNA and the carbonyl of the compound, alkyl-like interactions between the arginine (B:545) of the enzyme with the aromatic ring of the quinolone and the cyclohexane of the carbazole, van der Waals interactions between the proline (B:700) and the quinoline ring, and also between lysine (B:542) and the ethyl group of the compound (Figure 1C). The drug–receptor interaction was directed to the central part of the enzyme in subunit A (Figure 1D), an essential subunit for the cell replication process, which could affect the replication process of the genetic material [10,18]. On the other hand, the molecule has almost entirely hydrophobic regions (Figure 1C), which benefits the chemical structure, promoting greater specificity and efficiency [18]. These results suggest a possible interaction between the FQB-1 compound and topoisomerase II. Additionally, molecular docking of etoposide was performed as it is an inhibitor of human topoisomerase IIα (suppl Figure 1), which yielded a score of −11 kcal/mol, finding conventional interactions of hydrogen bonds or pi-sulfur related to amino acids of the enzyme such as methionine (B:1055), serine (B:1052), or arginine (B:1093), and also interacting with DNA nucleic acids such as guanine (E:1475), adenine (E:1474), and thymine (D:1457), among other interactions.

### 2.2. Cytotoxicity and Anti-Proliferative Effect of the FQB-1 Compound in Lewis Lung Carcinoma Cells

**Figure 2:**
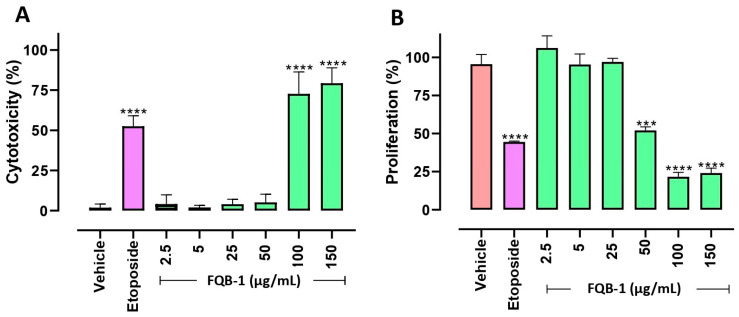
Cytotoxicity and anti-proliferative effect of the FQB-1 compound in Lewis lung carcinoma cells. (**A**) Percent cytotoxicity induced by FQB-1 compound was assessed by trypan blue exclusion staining in LLC cells. (**B**) Percentage of the antiproliferative effect of the FQB-1 compound using crystal violet staining in LLC cells. Both experiments were treated for 24 h at 37 °C with 5% CO_2_ in three independent experiments in triplicate, where 0.5% DMSO was used as a vehicle (negative control), etoposide at 30 µg/mL as a positive control, and increasing concentrations of FQB-1 were assessed. The statistical analysis of one-way ANOVA was carried out with a Tukey post hoc, where the experimental groups were compared with the vehicle; it was considered significant: *** *p* < 0.001, **** *p* < 0.0001.

Based on the favorable results of the prediction obtained from molecular docking, we focused on evaluating the FQB-1 compound in Lewis lung carcinoma (LLC) cells in a concentration–response curve at increasing concentrations of 2.5, 5, 25, 50, 100, and 150 mg/mL; 0.5% DMSO was the vehicle, and etoposide at 30 mg/mL was used as the positive control. In this evaluation, it was found that the vehicle presented an average basal cytotoxic activity of 2%, and the etoposide showed an average cytotoxicity of 50%. Meanwhile, the FQB-1 compound presented a significantly greater cytotoxic effect than the positive control etoposide, reaching 73% from 100 µg/mL (Figure 2A).

The antiproliferative effect of the compound FQB-1 was evaluated in the LLC cells. The proliferation control (vehicle) showed an average of 97%, etoposide-treated cells showed a decrease in average proliferation of 49%, and interestingly, the compound FQB-1 (50 µg/mL) showed a comparable effect to etoposide. Then, increasing concentrations of FQB-1 revealed an effect at 50, 100, and 150 µg/mL, which caused a statistically significant decrease in cell proliferation (Figure 2B).

### 2.3. FQB-1 Decreases Tumor Growth in Lung Carcinoma of C57BL/6 Strain Mice

**Figure 3:**
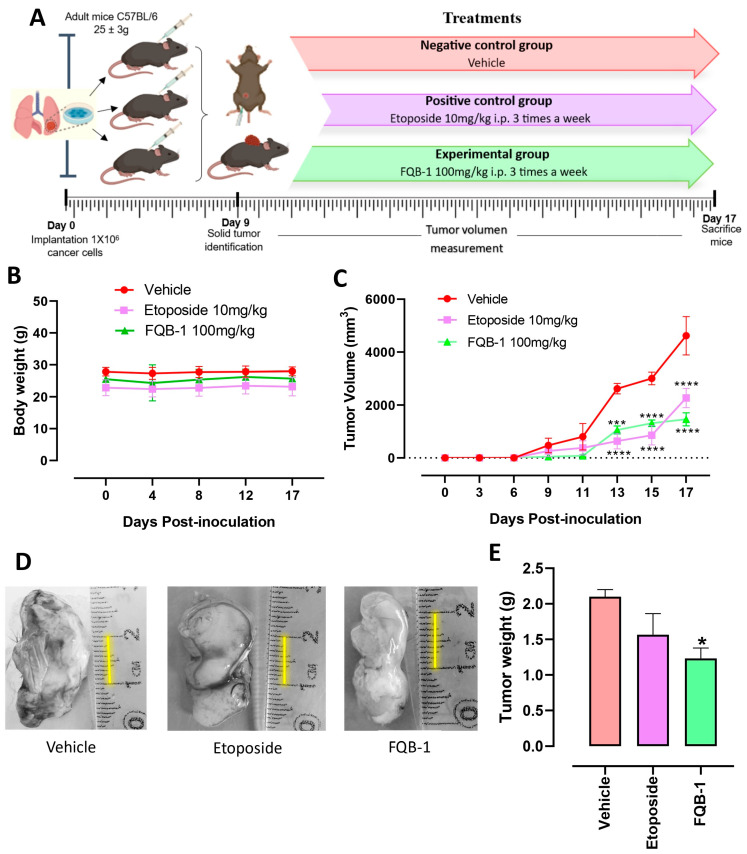
The compound FQB-1 decreases tumor growth in lung carcinoma of C57BL/6 strain mice. (**A**) Treatment scheme in C57BL/6 strain mice of both sexes weighing 25 ± 3 g; inoculated with 1 × 10^6^ LLC cells on the back of the mice. (**B**) The weight of the mice was monitored from the beginning of the experiment until their sacrifice. (**C**) Once the presence of the solid tumor was observed, the mice were randomized and separated into 3 experimental groups, made up of 4 mice per group. Tumor volume in mm^3^ was calculated using the Attia–Weiss formula. The treatment scheme was administered three times a week intraperitoneally in the three experimental groups. The start of treatment began the day after the appearance of the tumor (day 6 post-inoculation) as follows: a vehicle group (water), a group with etoposide at 10 mg/kg, and an experimental group with FQB-1 at 100 mg/kg. On day 17, the mice were euthanized by an overdose of the anesthetic pentobarbital administered intraperitoneally. The mean ± standard deviation of each experimental group was plotted, and comparisons of the tumor’s growth in the FQB-1 and etoposide groups versus the vehicle group were made. A two-way ANOVA statistical analysis was carried out, with Sidak’s post hoc test used to determine significant differences (*** *p* < 0.001, **** *p* < 0.0001). (**D**) Representative images by treatment group of the resected tumors. (**E**) The average weight of tumors removed per experimental group is shown as mean ± standard deviation. A one-way ANOVA with Tukey post hoc was performed as a statistical analysis; the etoposide group and FQB-1 were compared with the vehicle, taking the differences as significant * *p* < 0.05.

We worked with adult mice of the C57BL/6 strain of both genders, weighing 25 ± 3 g, which were used for our syngeneic lung adenocarcinoma model. The animals were inoculated subcutaneously with 1 × 10^6^ LLC cells on the back of each mouse and were monitored daily until the presence of the solid tumor was detected to start treatment intraperitoneally (Figure 3A). Animals were randomly assigned to each treatment group (*n* = 4/experimental group). The experimental groups were the vehicle group (administered with sterile water), the positive control group (treated with etoposide 10 mg/kg), and the third group treated with FQB-1 at a dose of 100 mg/kg. Body weight was recorded from the beginning of cell inoculation on the back of the mouse until day 17 of the sacrifice of the animals. All experimental groups maintained a constant body weight (no significant changes in body weight) from the beginning to the end of treatment, which indicates that despite the presence of the tumor and everything that the pathology entails, the treatment tolerability in our preclinical model with a frequency medium of administration with durable responses was relevant to follow up; therefore, etoposide and FQB-1 were well tolerated (Figure 3B).

After inoculation of 1 × 10^6^ LLC cells on the back of the mouse, the tumors were detected on day 9 post-implantation, followed by the measurement of the width and length of the tumor to determine the volume, recorded every third day until the end of treatment. The tumor growth in the group without treatment was practically exponential, in contrast with the groups that were treated with etoposide and FQB-1 (Figure 3C). When tumors reached day 13 post-implantation, significant differences began to be observed with both the administration of etoposide and the compound FQB-1 compared to the group without treatment. These results indicate a reduction in tumor growth in mice treated with etoposide or FQB-1. This shows that the compound FQB-1 presents an antitumor effect in the murine model of xenograft carcinoma comparable to the effect shown by the standard of care etoposide.

Once the treatment was completed, the mice were euthanized, and the tumors were removed, photographed (Figure 3D), and weighed. The tumors in the group without treatment reached an average weight of 2.1 g, while in the treated groups, a decrease in tumor weight was observed. In the etoposide-treated group, the tumors reached an average weight of 1.56 g; however, the tumor’s weight was not statistically different than the control group, unlike the group treated with the compound FQB-1, where the decrease in tumor weight was statistically significant, with a weight average of 1.06 g compared to the untreated group (Figure 3E).

### 2.4. FQB-1 Decreases Mitosis and Increases Necrosis in Lewis Lung Carcinoma Tumors

**Figure 4:**
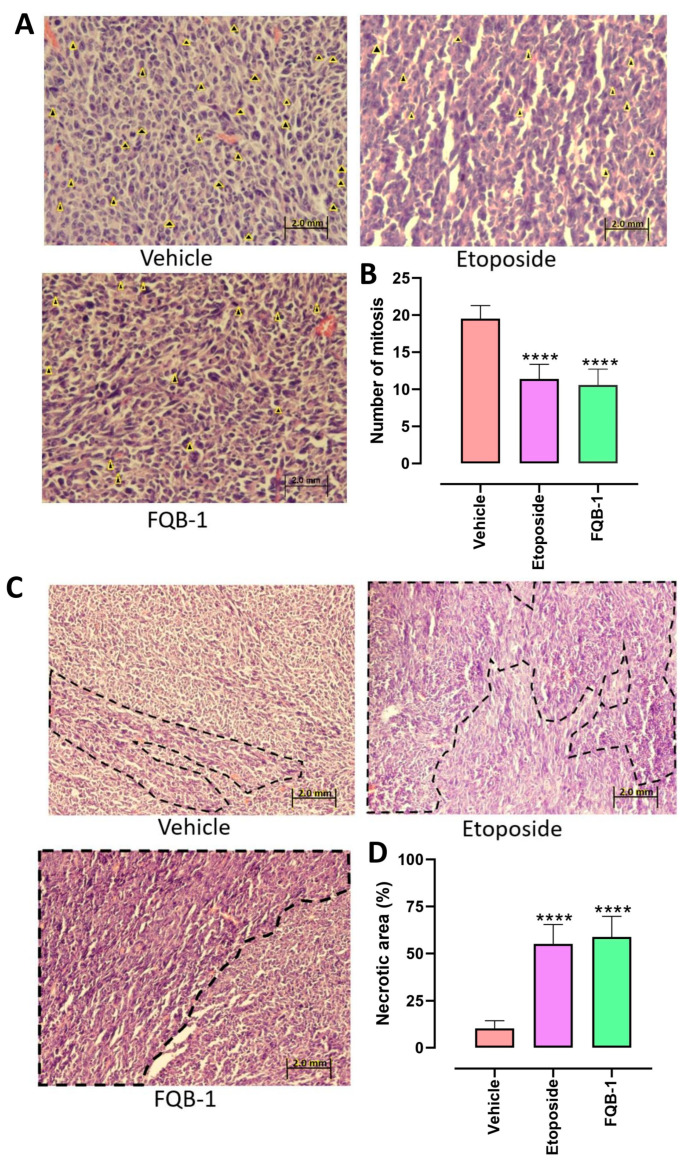
The compound FQB-1 decreases mitosis and increases necrosis in Lewis lung carcinoma tumors. (**A**) Representative images of 4 µm sections of tumors excised from C57BL/6 strain mice stained with hematoxylin–eosin, captured at 60× magnification. Arrowheads indicate cells undergoing mitosis in each experimental group. (**B**) The number of cells in the process of mitosis observed in 30 fields per experimental group was counted: vehicle (water), etoposide (10 mg/kg), and FQB-1 (100 mg/kg). The graph shows the mean ± standard deviation. The experimental groups etoposide and FQB-1 were compared with the vehicle, using one-way ANOVA followed by a Tukey post hoc **** *p* < 0.0001. (**C**) Representative images of 4 µm thick sections of tumors excised from C57BL/6 strain mice stained with hematoxylin–eosin, captured at 40× magnification. (**D**) The percentage of the necrotic area was determined with the 15-field Image J program for each experimental group. The values were graphed with mean ± standard deviation. The etoposide (10 mg/kg) and FQB-1 (100 mg/kg) experimental groups were compared with the vehicle. One-way ANOVA was performed with a Tukey post hoc **** *p* < 0.0001.

The excised tumors were processed and stained with hematoxylin and eosin for histopathological analysis, where the cells undergoing a mitotic process were counted. The four tumors per experimental group were examined, and the values of 30 fields were averaged under a 60X objective of a microscope. A representative image of each group was selected to show the mitotic cells marked with yellow arrows (Figure 4A). In the group without treatment, an average of 19 cells in the process of mitotic division per field was observed, in contrast to the groups treated with etoposide and FQB-1, where a significant decrease in cells in mitosis was observed, reaching an average of 11 mitoses per field (Figure 4B).

Necrotic areas determined tumor viability; the group without treatment presented an average of 10% necrosis. In parallel, the groups treated with etoposide and FQB-1 showed an increase in tumor necrosis, exceeding 50% of necrotic areas per field (Figure 4C,D). These results validate the potential for FQB-1 to compromise tumor integrity.

## 3. Discussion

Cancer is a complex disease with high morbidity and mortality rates worldwide. As such, the search for effective therapeutic targets is essential for the development of new drugs. Among these, the inhibition of human topoisomerase IIα has shown encouraging results against cancer treatment, as exemplified by etoposide, a first-line drug for lung cancer [19]. However, etoposide has been in use for over four decades, leading to reduced efficacy [20]. Thus, identifying new alternatives that counteract this problem is the goal of this research. Fluoroquinolones have emerged as a promising class of drugs with potential antitumor activity [8].

In this context, the fluoroquinolone derivative FQB-1 was evaluated for its anti-tumor potential. FQB-1 has a stable interaction with the human topoisomerase IIα 5GWK, as reported by Veyna-Hurtado L [21], who evaluated the in silico interactions of a borated quinolone with bacterial gyrase, finding similar interactions to those shown here. FQB-1 exhibited a docking score of −9.8 kcal/mol, comparable to that of etoposide (−11 kcal/mol), and demonstrated stronger binding affinity and stability than other fluoroquinolones, including ciprofloxacin, enoxacin, gatifloxacin, lomefloxacin, moxifloxacin, norfloxacin, and sarafloxacin, which scored from −6 to −7 kcal/mol [22]. These results suggest that FQB-1 may exert its cytotoxic effects through enzyme inhibition and triggering cell death pathways, as previously described by Strober et al. [23]. In general, the efficacy of topoisomerase-targeting drugs is closely associated with their ability to stabilize enzyme–DNA complexes, leading to DNA damage and genomic instability [24].

The compound FQB-1 induces significant cytotoxicity in LLC cells. This is due to the structural conformation of the molecule with substitution at C-3 with the difluoroboryl group, which, being a densely electronegative group, inducing electronic distortion on the molecule, and causing multiple interactions with amino acids of the enzyme [10], and furthermore, from the careful addition of a nucleophile such as carbazole at C-7, which also contributes to the interaction with the enzyme inducing the inhibition of IIα topoisomerase; other authors such as Laza-rević and collaborators [25] and Ahmadi et al. [26] introduced metals such as Au, Zn, and Ru to quinolones and evaluated their cytotoxic effect on A549 lung cancer cells and MCF-7 of breast cancer cells, where they obtained significant cytotoxic results at concentrations of 50–800 µg/mL for 72 h. The fluoroquinolone levofloxacin and derivatives have shown cytotoxic effects in combination with doxorubicin in the A549 cell line, a non-small cell lung cancer (NSCLC) cell line, in addition to antioxidant effects by inhibiting aldehyde dehydrogenase (ALDH) [27]. Synthesized levofloxacin-based compounds show cytotoxic effects by inhibiting topoisomerase IIβ in breast (MCF7), liver (Hep3B), and leukemia (L-SR) cancer cells [28]. Moreover, a screen with 19 fluoroquinolone analogues, including ciprofloxacin, moxifloxacin, and ofloxacin, revealed cytotoxic effects in multiple human cancers [29]. Two compounds (IIIf and VIb) were particularly effective in reducing cell viability, including cancer cells from lungs, leukemia, colon, breast, renal, central nervous system, ovarian, prostate, and melanoma [29].

Compared to these compounds, FQB-1 stands out for its efficacy at lower concentrations and shorter exposure times, producing robust cytotoxic effects. Its antiproliferative activity was also confirmed through cell proliferation assays. These results are consistent with findings by Yadav et al. [8], who demonstrated that fluoroquinolones combined with copper or zinc (e.g., levofloxacin–Cu, ofloxacin–Zn, norfloxacin–Zn) significantly inhibited proliferation in MCF-7 and HeLa cancer cells.

In our study, the incorporation of a difluoroboryl group at the C-3 position markedly enhanced both cytotoxic and antiproliferative effects. Furthermore, *in vivo* experiments have supported the anti-tumor activity of FQB-1. While previous studies with 2-quinolone derivatives showed limited efficacy in Ehrlich ascites carcinoma [30] and MCF-7 breast cancer models unless combined with other drugs like etoposide [31], our results show that FQB-1 alone, at a dose of 100 mg/kg, produced a significant antitumor response. This performance is superior to earlier quinolone derivatives, which often lacked standalone efficacy. In addition to reducing tumor growth, FQB-1 significantly decreased mitotic activity and increased necrosis in tumor cells. These findings align with those reported by Cuellar et al. (2023), who observed similar mitotic and necrotic changes following etoposide treatment in LLC cells [32]. Overall, our data strongly support the potential of FQB-1 as a potent antitumor agent and encourage continued investigation into its molecular mechanisms and therapeutic applications.

## 4. Materials and Methods

### 4.1. Synthesis of Difluoroboroyl 1-Ethyl-7-(1,2,3,4-tetrahydro-9H-carbazol-9-yl)-6-fluoro-4-oxo-1,4-dihydroquinolin-3-carboxylate (FQB-1)

Synthesis was carried out using the method reported by Hernández-López et al. [13], which consists of five reaction steps: The first reaction step consists of the formation of the diethyl 2-[(3,4-difluorophenylamino)methylene]malonate 2 from the condensation between the 3,4-difluoroaniline 1 and diethyl etoximethylenmalonate (EMME), with a of 83% reaction yield and a melting point (m.p.) of 70–71 °C, which was confirmed by 1H NMR (in DMSO-d6); two chemical shifts were observed at 1.33 and 1.38 ppm, corresponding to two triplet signals (3J= 7.00 Hz) of the methyl groups, as well as two quartet signals at 4.25 and 4.31 ppm (3J = 7.00 Hz) due to the methylenes of the ester group, while three signals of aromatic hydrogens were observed at 6.85 ppm (dd, 3JHF = 9.00 Hz, 4JHF = 6.50 Hz), 6.98 ppm (m) and 7.17 ppm (dd, 3JHF = 9.00 Hz, 4JHF = 6.50 Hz), vinyl hydrogen presented a chemical shift to 8.36 ppm (d, 3J = 13.50 Hz) and amine hydrogen 10.97 ppm (d, 3J = 13.50 Hz).

The second reaction step involved the formation of the quinoline ring through an intramolecular thermocyclization reaction with diphenyl ether at 250–260 °C, generating the 6,7-difluoro-4-oxo-1,4-dihydroquinoline-3-ethyl carboxylate (3) with an 85% yield and m.p. of 270–271 °C, which, in 1H NMR only showed a triplet signal at 1.27 ppm (3J = 7.00 Hz), a quartet signal 4.21 ppm (3J = 7.00 Hz), and two aromatic hydrogen signals 7.64 ppm (dd, 3JHF = 8.96 Hz, 4JHF = 7.44 Hz) and 7.99 ppm (dd, 3JHF = 8.96 Hz, 4JHF = 7.44 Hz), which confirms the intramolecular thermocycling reaction, additionally the singlet signal of vinyl hydrogen to 8.59 ppm moving to the low field due to the increase in the anisotropic effect of the quinolone ring formed. In the third reaction step, the incorporation of the ethyl group in N1 of the quinolone ring was carried out by using iodoethane, DMF, and K_2_CO_3_ for obtaining the 1-ethyl-6,7-difluoro-4-oxo-1,4-dihydroquinoline-3- ethyl carboxylate (4) with a reaction yield of 85% and m.p. 140–141 °C, confirmed by 1H NMR, where the signals of the ethyl ester were observed (1.27 ppm, t, 3J = 7.28 Hz and 4.22 ppm, c, 3J = 7.28 Hz) and ethyl amine (1.33 ppm, t, 3J = 7.28 Hz and 4.37 ppm, c, 3J = 7.28 Hz), as well as a multiplet signal in the aromatic hydrogen region at 8.06 ppm and a singlet signal at 8.69 ppm for vinyl hydrogen. To favor the regio-selective SNA at carbon 7 (C-7) of the fluoroquinolone ring, the fourth step of the reaction was carried out with the formation of the fluoroquinolone–boron complex, for which, by using trifluoroboryl diethyl etherate (BF3·OEt2) in diphenyl ether a 200 °C, was obtained from 1-ethyl-6,7-difluoro-4-oxo-1,4-dihydroquinoline-3-carboxilate of difluoroboryl 5, with a reaction yield of 77% and a m.p. 262–264 °C, observing by 1H NMR of the amino ethyl a 1.48 ppm (t, 3J = 7.32 Hz) and 4.88 ppm (c, 3J = 7.32 Hz), aromatic hydrogens 8.55 ppm (dd, 3JHF = 12.44 Hz, 4JHF = 6.59 Hz) and 8.69 ppm (dd, 3JHF = 12.44 Hz, 4JHF = 6.59 Hz), vinyl hydrogen at 9.61 ppm; while the presence of boron difluoride was determined indirectly by 19F NMR, observing the chemical shifts of the fluorine atoms in the quinoline ring at −121.37 ppm (d, 3JFF = 23.05 Hz) and −132.31 ppm (d, 3JFF = 23.05 Hz); while the two fluors bound to boron were observed as a singlet signal at −140.33 ppm, confirming the formation of the product. Finally, the following reaction was made: 1,2,3,4-tetrahydro-9H-carbazole, triethylamine (TEA), dimethylsulfoxide (DMSO), and compound 5 at 100 °C to obtain FQB-1 with a 39% yield and a m.p. of 293–300 °C. The chemical structure was confirmed by 1H NMR, observing the chemical shifts of the hydrogens present in the quinolone ring at 1.41 ppm (d, JH-H = 6.36 Hz), 3.90 ppm (m, 2H), 7.35 ppm (d, 3JHF = 8.32 Hz, 1H), 8.14 (d, 4J = 7.16 Hz), and 8.90 (s). The hydrogens present in the ring of 1,2,3,4-tetrahydro-9H-carbazole-9-yl, bound at C-7 of the fluoroquinolone at 7.22 ppm (t, 3J = 5.96 Hz, 1H), 6.92 ppm (m, 3H), 3.72 ppm (m, 1H), 2.81 ppm (m, 1H) and a 2.26 ppm (d, 3J = 11.52 Hz, 2H) can be seen in Figure S1. Additionally, the progress of the reaction and the purity of the intermediate products obtained in each step of the synthetic route were monitored by aluminum thin-layer chromatography impregnated with silica gel. (Sigma-Aldrich, cat no. 60778-25EA) and confirmed with 1H, 13C, and 19F NMR, in a “Bruker Ascend 400 MHz NMR spectrometer”.

### 4.2. Molecular Docking

The prediction calculation using molecular docking predicts the ligand–receptor interaction energies based on ∆G (Gibbs free energy), which represents the energy of a system that is available in its environment [33]. In this analysis, we worked with the methodology reported by Pham TDM and collaborators [21] based on the enzyme recovered from the protein data bank (PDB) database, human IIα topoisomerase co-crystallized with etoposide with the code 5GWK, which reports a resolution of 3.14 Å. Using the Avogadro 1.2.0 software, the ligand was subjected to modifications to favor the interaction points and direct the study to a standard range of the enzyme. For this, the chemical structure was optimized, the energy was minimized, and the modifications that favored the binding of the enzyme were added. The crystallized ligands and solvents were eliminated to prevent interference with the calculations performed. The Chimera version 1.18 and AutoDock Tools software version 1.5.7 programs were used to prepare the receptor. The Mg^2+^ ion was considered because it is a metal ion, which allows for a chelation reaction. The compound was designed in the ChemDraw 8.0 program, and the structure was optimized to obtain the most favorable position to interact with the receptor. For this, the Avogadro program was used to obtain the three-dimensional coordinates to which the interaction was directed. Finally, FQB-1 was docked with IIα topoisomerase using AutoDock Vina, obtaining a series of results visualized in Discovery Studio Visualizer.

### 4.3. Cytotoxicity Evaluation

The Lewis lung carcinoma cell line (LLC, ATCC CRL-1642) was cultured in DMEM medium BIO-L0060-500, Biowest S.A.S., Nuaillé, France) supplemented with 1% antibiotic (penicillin with streptomycin, L0022-100, Biowest S.A.S., France), and 15% serum newborn calf serum (SNB) (S0750-500, Biowest S.A.S., France). LLC cells were cultured in an incubator at 37 °C with 5% CO_2_ until a confluence of 80% was reached and then treated for 24 h in 24-well plates. The evaluations were carried out in triplicate in three independent experiments. After the stimulation time, the cells were trypsinized (BIO-L0931-500, Biowest S.A.S., France) and subsequently stained with trypan blue (Sigma-Aldrich, St. Louis, MO, USA), mounted in a Neubauer chamber, and observed under a microscope to count viable (refringent) and non-viable (stained) cells [23]. The treatments were the following: vehicle (0.5% DMSO), positive control (etoposide 30 µg/mL, Tosuben, PiSA Laboratories, Mexico City, Mexico), and FQB-1 compound (2.5, 5, 25, 50, 100, and 150 mg/mL) which is a potent inhibitor of topoisomerase II, as well as fluoroquinolones [20]. This similarity in mechanism of action allows for comparison if both drugs to act on the same pocket of the pharmacological target, which can interfere with the ligand–receptor interaction, or if the difference in pocket favors the efficacy [13]. On the other hand, etoposide is currently administered in the clinic for the treatment of lung carcinoma, so its inclusion allows us to validate the efficacy of the compound because its use has been widely documented in patients with this disease [20].

### 4.4. Cell Proliferation Assay

Cell proliferation was assessed following a previously described protocol [34]. After 24 h of treatment with the test compounds in a 24-well plate, as outlined in the cytotoxicity evaluation, the supplemented DMEM medium was removed. Cells were then fixed with absolute methanol and stained with 0.4% crystal violet (Sigma-Aldrich). Following staining, the excess dye was discarded, and the wells were rinsed to remove unbound crystal violet. Acetic acid was added to solubilize the bound dye, and the resulting suspension was transferred to a 96-well plate. Absorbance was measured at 600 nm using a Thermo Scientific Multiskan Sky microplate spectrophotometer.

### 4.5. Syngeneic Lung Carcinoma Model

We worked with male and female C57BL/6 adult immunocompetent mice (25 ± 3 g), maintained with water and food ad libitum, under a 12/12 h light/darkness cycle and controlled temperature (20–22 °C). All mice were habituated for a week before handling. The experiments were carried out following our Institutional Internal Ethics Committee for the Care and Use of Laboratory Animals (ACS/UAZ/362/2023), which complies with the Mexican Official Norm (NOM-062-ZOO-1999) and the ARRIVE guidelines. All mice were inoculated with 1 million LLC tumor cells (C57BL/6 with the same genetic background as LLC cells) on the back of the mouse as previously described [32,35,36], and day 0 was used as the day of cell inoculation. After inoculation, the body weight and the presence of the solid tumor were monitored daily. Once the tumor was detected, tumor measurement began, and the treatment was administered intraperitoneally (100 mg/kg, to a final volume of 0.2 mL) three times per week. The length and width of the tumor were measured using a caliper, and the tumor’s volume was calculated using the formula V = width × length^2^ × π/6 [36,37]. Tumor volume was recorded every third day, each experimental group consisted of 4 mice, to which a treatment scheme was followed as shown in Figure 3A. (a) Negative control group: administration of purified water; (b) positive control group: purified water + etoposide 10 mg/kg, 3 times per week; (c) experimental group: purified water + FQB-1 at a dose of 100 mg/kg, 3 times per week.

### 4.6. Histopathological Analysis

The removed tumors were preserved in 10% formalin, and subsequently, the tissues were dehydrated in an increasing gradation of ethanol ranging from 50° to 100°, then a wash was performed with xylene to continue with the paraffin passage in an incubator at 60 °C; once the three repetitions had elapsed, the block was formed with liquid paraffin, supported by a mold to cool and unmold it. The tissues were sectioned at a thickness of 4 mm and passed through a flotation bath to extend the tissues and collect them with slides for subsequent staining with hematoxylin and eosin, starting with deparaffinization with passes through xylene, and then the tissues were hydrated with ethanol at decreasing concentrations. Then the sections were stained with hematoxylin followed by eosin, and finally, the sections were dehydrated with a series of ethanol at increasing concentrations and rinsed with xylene. The sections were fixed with resin and analyzed in the microscope.

Excised tumor tissues were fixed in 10% neutral-buffered formalin. Following fixation, the samples were dehydrated through a graded ethanol series (50% to 100%), cleared in xylene, and embedded in paraffin at 60 °C. After three paraffin exchanges, tissue blocks were formed by pouring molten paraffin into molds, which were then cooled and demolded.

Paraffin-embedded tissues were sectioned at a thickness of 4 µm using a microtome. The sections were transferred to a warm water flotation bath to flatten, then mounted onto glass slides. For histological evaluation, sections underwent deparaffinization in xylene, followed by rehydration through a descending ethanol gradient. Hematoxylin and eosin (H&E) staining was performed, beginning with nuclear staining using hematoxylin, followed by counterstaining with eosin. Finally, the sections were dehydrated through an ascending ethanol series, cleared in xylene, and mounted with resin for microscopic examination.

### 4.7. Quantification of Mitotic Cells and Analysis of Necrotic Areas

To count cells in mitosis, 30 fields per experimental group were quantified at a 60× objective. The analysis of necrotic areas was performed using Image J, where fifteen fields per group were delimited and analyzed under a 40× objective.

### 4.8. Statistical Analysis

The data were analyzed with the Shapiro–Wilk test to determine normality. The data were obtained with normal distribution. For the evaluations of cytotoxicity and proliferation, mitosis, and necrosis, they were analyzed by one-way ANOVA followed by Tukey’s post hoc test. Tumor growth data were analyzed by two-way ANOVA followed by Sidak’s post test. All statistical analysis was performed in GraphPad Prism (8.0.2) software.

Data normality was assessed using the Shapiro–Wilk test, confirming a normal distribution for all datasets. Comparisons of cytotoxicity, proliferation, mitotic index, and necrosis were conducted using one-way ANOVA followed by Tukey’s post hoc test for multiple comparisons. Tumor growth data were analyzed using two-way ANOVA with Sidak’s multiple comparison post test. All statistical analyses were performed using GraphPad Prism software (version 8.0.2; GraphPad Software, San Diego, CA, USA). A *p*-value of <0.05 was considered statistically significant.

## 5. Conclusions

The compound derived from fluoroquinolone FQB-1 demonstrates stable interaction with human topoisomerase IIα in molecular docking studies (score −9.8 kcal/mol). In biological models, FQB-1 induces cytotoxic and anti-proliferative effects against LLC cells and significantly reduces tumor growth by decreasing mitotic activity and compromising tumor viability (comparable to the effects observed with etoposide). This initial investigation highlights the potential of this fluoroquinolone derivative against an aggressive syngeneic tumor model, encouraging further studies with FQB-1. As perspectives, we will expand fluoroquinolone studies at the molecular level to confirm direct inhibition of topoisomerase using molecular biology techniques. We also aim to explore other possible mechanisms of action reported for other fluoroquinolones. Furthermore, screening FQB-1 on different tumor cells and conducting in vivo pharmacological studies will be essential to better understand the therapeutic potential of this compound.

## Data Availability

Data are contained within the article and Supplemental Figure.

## Supplementary Materials

The following supporting information can be downloaded at: https://www.mdpi.com/article/10.3390/ph18050664/s1. Figure S1: Two-dimensional representation of the amino acids of IIα human topoisomerase (5GWK) with etoposide.

## Abbreviations

FQ	Fluoroquinolones
DMSO	Dimethylsulfoxide
LLC	Lewis lung cancer
PDB	Protein data bank
