# Supplementary material for: Norfloxacin Derivative with Carbazole at C-7 FQB-1 Induces Cytotoxic, Antiproliferative, and Antitumor Effects in an Experimental Lung Carcinoma Model
Source: Pharmaceuticals (Basel). 2025 Apr 30;18(5):664. doi: 10.3390/ph18050664 (PMC12114400; doi:10.3390/ph18050664)
Supplement: Supplementary file 1 [file pharmaceuticals-18-00664-s001.zip › pharmaceuticals-3558297-supplementary.pdf]

Supplementary Information

Norfloxacin derivative with carbazole at C-7 FQB-1 induces cy-totoxic, antiproliferative, and antitumor effects in an experimental lung carcinoma model.

Alondra Bocanegra-Zapata, Hiram Hernández-López, Socorro Leyva-Ramos, Rodolfo Daniel Cervantes-Villagrana, Marisol Galván-Valencia, L. Angel Veyna-Hurtado, Norma Guadalupe Ramírez Tovar, Damaris Albores-García , Juan Armando Flores de la Torre , Alberto Rafael Cervantes-Villagrana 3*

General Synthesis of difluroboryl 1-ethyl-7-(1,2,3,4-tetrahydro-9*H*-carbazol-9-yl)-6-fluoro-4-oxo-1,4-dihydroquinoline-3-carboxilate (**FQB-1**)

The synthesis of **FQB-1** was in accordance of methodology reported by Hernández-López et al (19). An example of the synthesis, the last step in the pathway to obtain **FQB-1**, is herein presented:

In a round-button flask was added 1.5 mL of DMSO, 69.4 µL (0.5 mmol) of triethylamine (TEA), 100 mg (332.85 µmol) of difluoroboryl 1-ethyl-6,7-difluoro-4-oxo-1,4-dihydroquinoline-3-carboxylate and 85.63 mg (0.5 mmol) of 1,2,3,4-tetrahydro-9*H*-carbazol in constant stirring for 20 to 25 hours at 80 ºC. The beginning brown slight turbidity observed inside of the reaction flask was turning to cream color indicating the reaction progresses. After time reaction, 1 mL of ethanol was added to the reaction mixture, obtaining a cream solid that was filtered by vacuum and washed with ethanol, obtaining **FQB-1** with a yield of 46 % and mp of 293-300 ºC (in a Fisher-Johns melting-point apparatus).

^{1}H NMR spectra (Figure 1S) was made in a 400 MHz Varian spectrometer apparatus in DMSO-*d6*, and TMS as internal standard, obtaining the following data: δ (ppm) 8.90 (*s*, 1H), 8.14 (*d*, $J_{H\text{-}F}$ = 7.16 Hz, 1H), 7.35 (*d*, $J_{H\text{-}F}$ = 8.32 Hz, 1H), 7.22 (*t*, $J_{H\text{-}H}$ = 5.96 Hz, 1H), 6.92 (*m*, 3H), 3.90 (*m*, 2H), 3.72 (*m*, 1H), 2.81 (*m*, 1H), 2.26 (*d*, $J_{H\text{-}H}$ = 11.52, 2H), 1.41 (*d*, $J_{H\text{-}H}$ = 6.36 Hz).

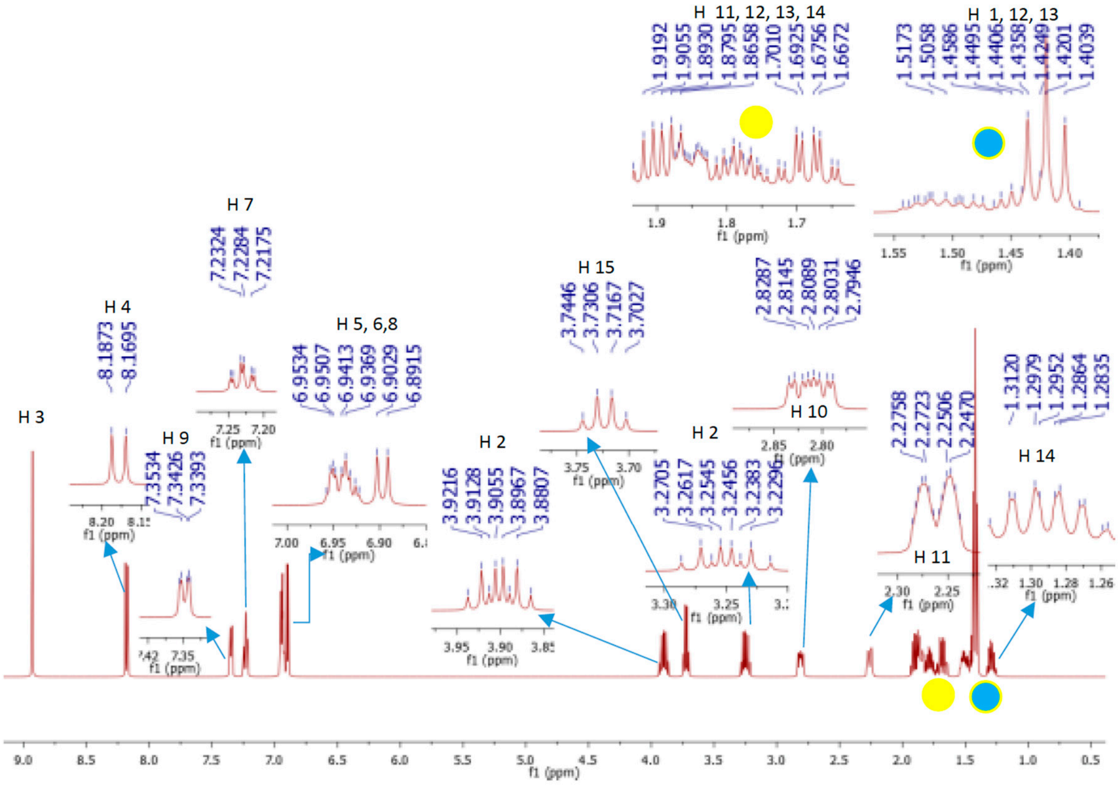

Figure S1. ^{1}H NMR spectra of **FQB-1** in DMSO-d_6 at 400 MHz.

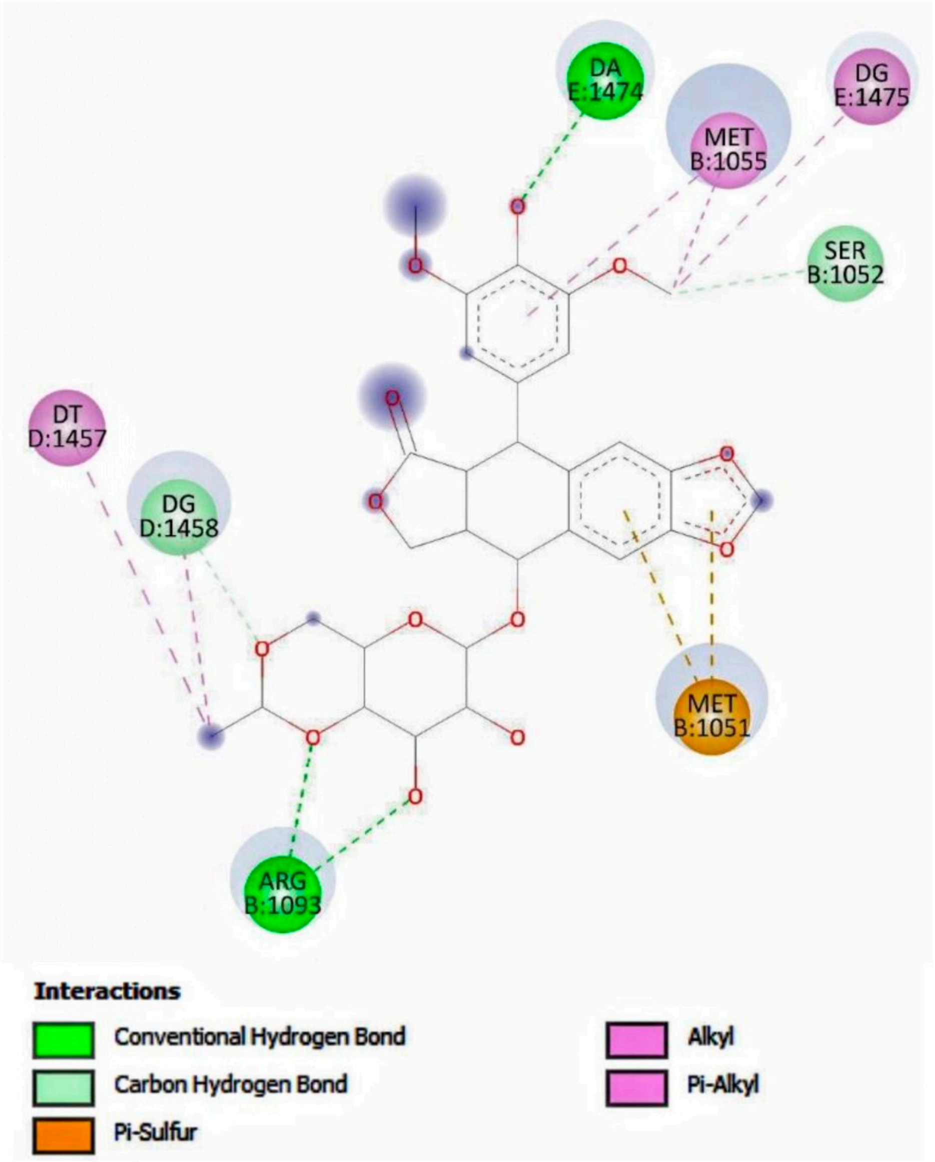

2D interactions representation of etoposide with human topoisomerase IIα.
